# Two single lung transplantations from one donor: lung twinning in the LAS era

**DOI:** 10.1186/s12931-024-02754-w

**Published:** 2024-03-18

**Authors:** Frank Langer, Philipp M. Lepper, Bettina Weingard, Parviz Aliyev, Robert Bals, Heinrike Wilkens

**Affiliations:** 1 Dept. of Thoracic Surgery, Saarland University Medical Center, 66424 Homburg / Saar, Germany; 2 Department of Internal Medicine V, Pneumology and Intensive Care Medicine, Saarland University Medical Center, Homburg / Saar, Germany

**Keywords:** Lung transplantation, Single lung transplantation, Lung twinning

## Abstract

**Objectives:**

The implementation of the Lung Allocation Score (LAS) in the Eurotransplant international collaborative framework decreased waiting list mortality, but organ shortage remains a significant problem. Transplantation of two single lungs from one donor into two recipients (lung twinning) may decrease waiting list mortality. We sought to analyze if this strategy can lead to an acceptable intermediate-term outcome.

**Methods:**

Since the LAS-implementation we performed 32 paired single-lung transplantations from 16 postmortal donors. Data and outcome were analyzed retrospectively comparing recipients receiving the first lung (first twins) with recipients receiving the second lung (second twins), left versus right transplantation and restrictive versus obstructive disease.

**Results:**

Survival at one year was 81% and 54% at five years. Veno-venous ECMO had been successfully used as bridge-to-transplant in three patients with ECMO-explantation immediately after surgery. Bronchial anastomotic complications were not observed in any patient. First twins and second twins exhibited similar survival (*p* = 0.82) despite higher LAS in first twins (median 45 versus 34, *p* < 0.001) and longer cold ischemic time in second twins (280 ± 83 vs. 478 ± 125 min, *p* < 0.001). Survival of left and right transplantation was similar (*p* = 0.45) with similar best post-transplant FEV1 (68 ± 15% versus 62 ± 14%, *p* = 0.26). Survival was similar in restrictive and obstructive disease (*p* = 0.28) with better post-transplant FEV1 (70 ± 15% versus 57 ± 11%, *p* = 0.02) in restrictive disease.

**Conclusions:**

Performing two single-lung transplantations from one donor can be performed safely with encouraging intermediate-term outcome and good functional capacity. Lung twinning maximizes the donor pool and may help to overcome severe organ shortage.

**Clinical trials:**

This research is not a clinical trial. Thus no registration details will be provided.

## Introduction

Since the Lung Allocation Score (LAS) was introduced in Germany in 2011 a decreased number of deaths among patients on the waiting list could be documented [1]. Nevertheless death on the waiting list remains a clinical challenge due to severe organ shortage since only 10–30% of donor lungs are judged suitable for transplantation [2]. Extended donor criteria have been suggested to overcome this dilemma and have become clinical reality in daily practice [3]. The clinical value of ex-vivo-lung perfusion for marginal donors remains to be defined, yet [4]. 

In our center we have followed a simple and cost-effective alternative. We have consistently performed single-lung transplantations (sLTx) for patients with pulmonary fibrosis since 1995. Moreover we have tried to perform sLTx in patients with COPD/emphysema once hyperinflation of the remaining native lung was prevented with prior lung volume reduction. When a single lung was allocated for a fibrosis patient with higher LAS, the remaining donor lung was frequently allocated to our center for a COPD-patient with lower LAS. With this strategy we have created a cohort of lung twins, i.e. single-lung-recipients from the same donor [5]. Such approach expands the donor pool and may reduce waiting list mortality. The drawback of such strategy is that the second organ is exposed to prolonged ischemic time with increased risk of ischemia/reperfusion injury and primary graft failure [2]. In the current investigation we report our medium-term results with “lung twinning”.

## Methods

Between January 2012 and December 2023, a total of 205 LTx (sLTx *n* = 117, double LTx *n* = 88) were performed in our center. All sLTx (*n* = 32) from the same donor (*n* = 16) were included in the current retrospective investigation. Follow-up was performed by our transplant outpatient clinics. Data collection for the current retrospective investigation was approved by the Saarland University Medical Center Transplantation Ethics Committee before data collection. All patients had signed informed consent for data collection and analysis before admission on the transplant waiting list. The study was conducted in compliance with the Declaration of Helsinki.

### Statistical analysis

Data were expressed as mean ± standard deviation unless otherwise specified. Statistical analysis was performed using standard software (SigmaStat, Systat). Normal distribution was assessed using the Kolmogorov-Smirnov-test. Comparisons were perfomed between groups (normally distributed continuous data: t-test, non-normally distributed continuous data: Mann-Whitney-U-rank-test, discrete data: Fisher´s exact test). Kaplan-Meier-analyses of survival were also calculated using standard software (Prism, Graphpad) - the log-rank test was used to compare the survival distributions.

## Results

Underlying disease was classified as restrictive (*n* = 18 / 56%) or obstructive (*n* = 12 / 38%) lung disease (Table 1: Demographic data and postoperative outcome). Two patients with prior sLTx bronchiolitis obliterans syndrome (BOS 6%) underwent sLTx of the native contralateral lung. Sex was distributed equally (male *n* = 16, female *n* = 16) among recipients—recipient age ranged from 38 to 67 years (mean 58 ± 7 years).

**Table 1:** Demographic data and postoperative outcome

Twin Pair No.	Age	Sex	Disease type	LAS	sLTx type	Outcome	Best FEV1 (%)	CLAD Stage
1	59 50	f f	REST BOS	55 43	left right	alive 107 mo died 4 d postop (sepsis)	72	0
2	62 63	f f	REST OBST	49 36	left right	alive 84 mo died 6 mo postop (sepsis)	72	0
3	56 53	m m	REST REST	90 34	right left	died 11 mo postop (sepsis) alive 84 mo	50 70	0
4	45 59	m m	REST OBST	88 38	left right	died 70 mo postop (rejection) alive 75 mo	79 58	0 1
5	63 50	m m	REST OBST	40 32	right left	alive 65 mo alive 65 mo	90 53	0 3
6	52 61	m m	REST OBST	44 34	left right	died 40 mo postop (stroke) alive 63 mo	89 63	0 2
7	63 64	m m	REST OBST	67 32	left right	alive 61 mo died 39 mo postop (sepsis)	89 73	0 0
8	53 61	m m	BOS REST	43 38	right left	died 2 mo postop (sepsis) died 42 mo postop (lung cancer in native fibrosis lung)	98	0
9	54 64	f f	REST OBST	61 33	left right	died 42 mo postop (pneumonia) alive 58 mo	59 65	0 0
10	38 56	m m	REST OBST	92 34	left right	alive 56 mo alive 56 mo	65 61	0 0
11	55 56	m m	REST REST	38 34	left right	died 48 mo postop (lung cancer in native fibrosis lung) died 6 mo postop (pulmonary embolism)	52 57	0 0
12	61 66	f f	OBST OBST	34 31	left right	alive 53 mo died 47 mo postop (CLAD)	54 62	0 3
13	61 64	f f	OBST OBST	33 34	left right	alive 39 mo alive 39 mo	59 52	0 0
14	67 63	m f	REST OBST	46 40	left right	died 15 mo postop (COVID) alive 33 mo	53 69	0
15	38 61	f f	REST REST	46 34	left right	alive 16 mo alive 16 mo	60 73	0 0
16	62 57	f f	REST OBST	37 33	left right	died 5 mo postop (sepsis) alive 14 mo	30	3

(REST: restrictive lung disease, OBST: obstructive lung disease, BOS: bronchiolitis obliterans syndrome, CLAD: chronic lung allograft dysfunction)

A high LAS (> 50) was present in 6 patients, median LAS was 38 (range 31–92). Three patients required veno-venous ECMO-support as bridge-to-transplant (5, 51 and 75 days, awake ECMO *n* = 2). ). Transplantation occurred after a median waiting list time of 145 days in the first twin group and 964 days in the second twin group. Data were compared with the waiting list time of 63 double lung transplant recipients (median 283 days), who underwent transplantation in the same time period: Waiting time was similar for first twins and double lung transplants (145 versus 283 days; *p* = 0.76), while waiting time tended to be longer in the second twin group (964 versus 283 days, *p* = 0.056).

All donor lungs were cadaveric organs from donors within the Eurotransplant cooperation. Donor age was 48 ± 16 years, donor TLC was 6.2 ± 1.2 L, mean ventilation time was 5 ± 4 days, median C-reactive protein (CRP) was 120 ± 74 mg/L, and mean Horovitz index (pO2/FiO2) was 415 ± 88 mmHg. Ten donors had a history of smoking, 8 donors had abnormal bronchoscopy findings (inflammation, pus, aspiration). The first implantation was always performed for the patient with the higher LAS. Ischemic time was significantly longer for the second lung (280 ± 83 vs. 478 ± 125, *p* < 0.001). In all three patients requiring ECMO-bridge-to-transplant the ECMO-system could be explanted in operating room immediately after transplantation. No postoperative primary graft failure was observed. There was no difference between first and second twin groups in terms of ICU length of stay (first twin group: median 5 days, second twin group: 7 days; *p* = 0.36) and cumulative invasive and non-invasive ventilation time (first twin group: median 26 h, second twin group: 24.5 h; *p* = 0.58).

**Fig. 1:**
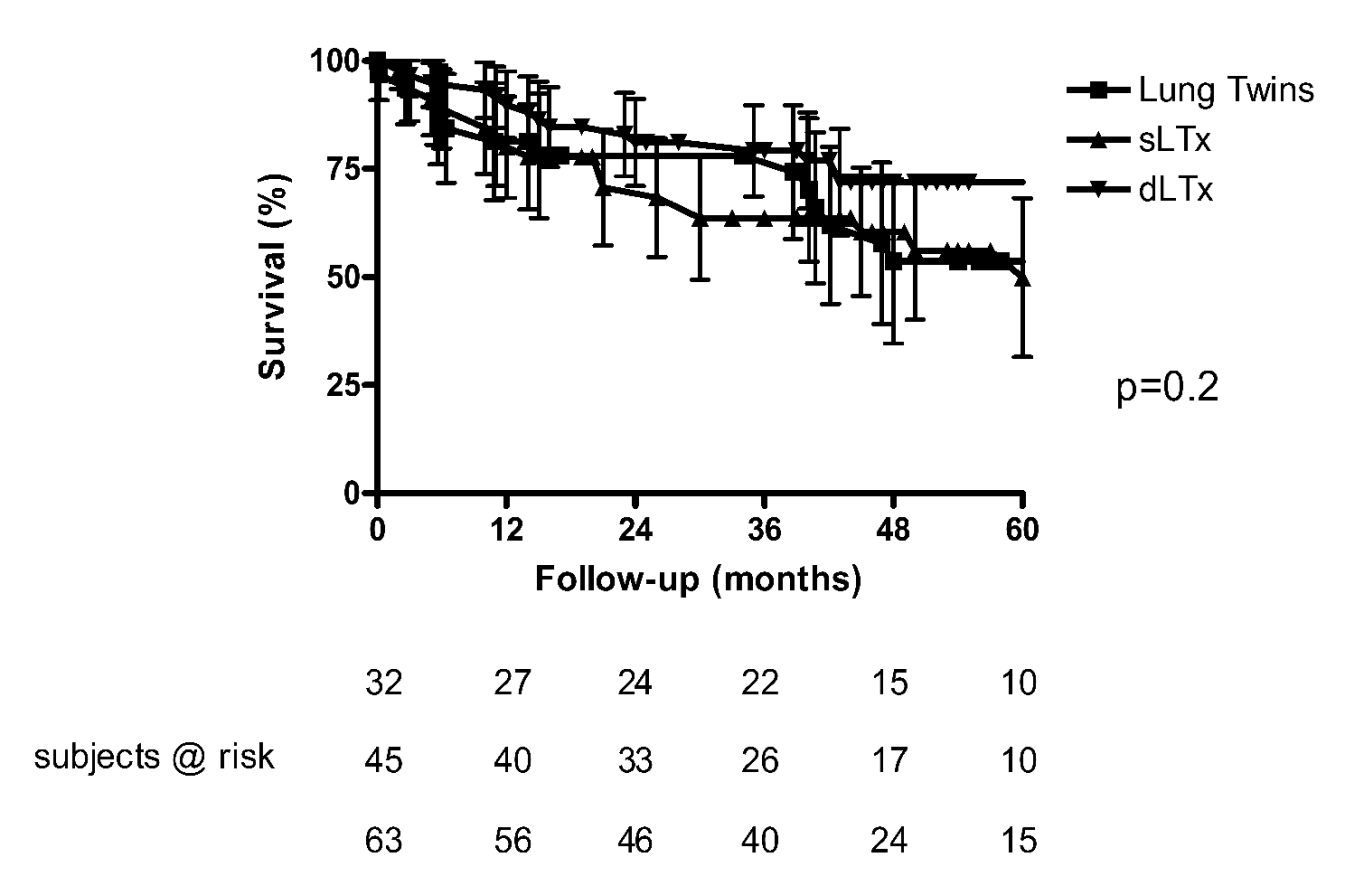
Kaplan-Meyer survival analysis: whole cohort

Post-transplant survival was 81% at one year and 54% at five years. Survival was similar compared with 45 standard sLTx (*p* = 0.71) and 63 double lung transplants (*p* = 0.2), which had been performed in our center in the same time period (Fig. 1). One BOS-patient died in hospital due to aspiration pneumonia. Median follow-up of the 31 remaining recipients was 42 months. Thirteen recipients died during the follow-up (pneumonia/sepsis *n* = 8, pulmonary embolism *n* = 1, lung cancer of native fibosis lung *n* = 2, acute rejection *n* = 1, stroke *n* = 1; Table 1: Demographic data and postoperative outcome). Surveillance bronchoscopy did not show any bronchial anastomotic complication such as dehiscence or stenosis.

### Subgroup analysis first twin versus second twin

**Fig. 2:**
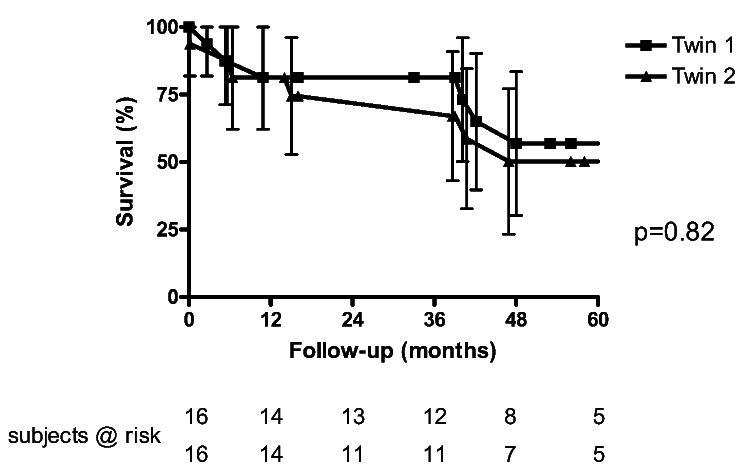
Kaplan-Meyer survival analysis: first twin versus second twin

Age was distributed similarly in recipients receiving the first lung (first twin) and patients receiving the second lung (56 ± 8 versus 60 ± 5, *p* = 0.11). First twin recipients had higher LAS (median 45 versus 34, *p* < 0.001) and had predominantely ILD (*n* = 13 / 81%). Cold ischemic time was longer in second twins (280 ± 83 vs. 478 ± 125, *p* < 0.001) Survival was similar in both goups (1-year-survival: 81 versus 81%, 5-year-survival: 57 versus 50%, *p* = 0.82; Fig. 2). Best postoperative FEV1 was similar in both groups (68 ± 14% versus 62 ± 15, *p* = 0.26). Eight surviving first lung recipients are in CLAD stage 0 – one patient has CLAD stage I. Five of nine surviving second lung recipients have no or mild (CLAD stage 0: *n* = 4, CLAD stage I: *n* = 1), while four patients have significant CLAD (CLAD stage II: *n* = 2, CLAD stage III: *n* = 2).

### Subgroup analysis left sLTx versus right sLTx

**Fig. 3:**
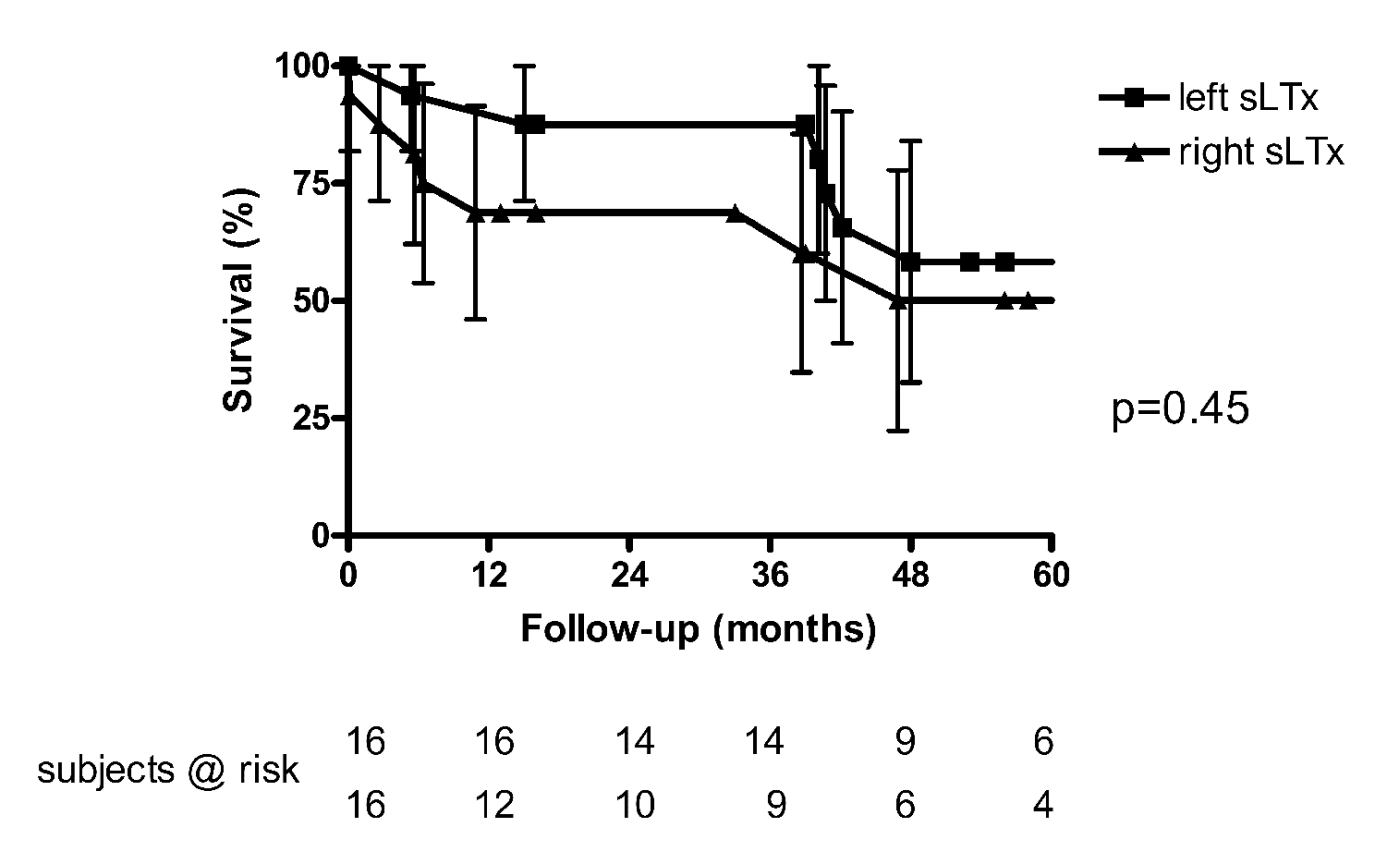
Kaplan-Meyer survival analysis: restrictive versus obstructive disease

Recipients receiving a left sided graft tended to be younger (56 ± 6 versus 60 ± 5, *p* = 0.06) and had a higher LAS (median 45 versus 34, *p* = 0.03). Underlying disease in left sLTx-recipients was predominantely restrictive (*n* = 13 / 81%). Survival tended to be superior in left lung allograft recipients after one year, but was similar in both groups in the further follow-up (1-year-survival: 94 versus 69%, 5-year-survival: 58 versus 50%, *p* = 0.45; Fig. 3). Best post-transplant FEV1 was similar in both subgroups (left sLTx: 68 ± 14% versus right sLTx: 62 ± 14%, *p* = 0.26). Eight recipients of the surviving recipients of a left lung allograft have no or mild CLAD (CLAD stage 0: *n* = 6, CLAD stage I: *n* = 2), while one patient has CLAD stage III. Six of nine surviving recipients of a right lung allograft hav no CLAD, while two patients have advanced CLAD (stage II: *n* = 1, stage III: *n* = 1).

### Subgroup analysis restrictive versus obstructive lung disease

**Fig. 4:**
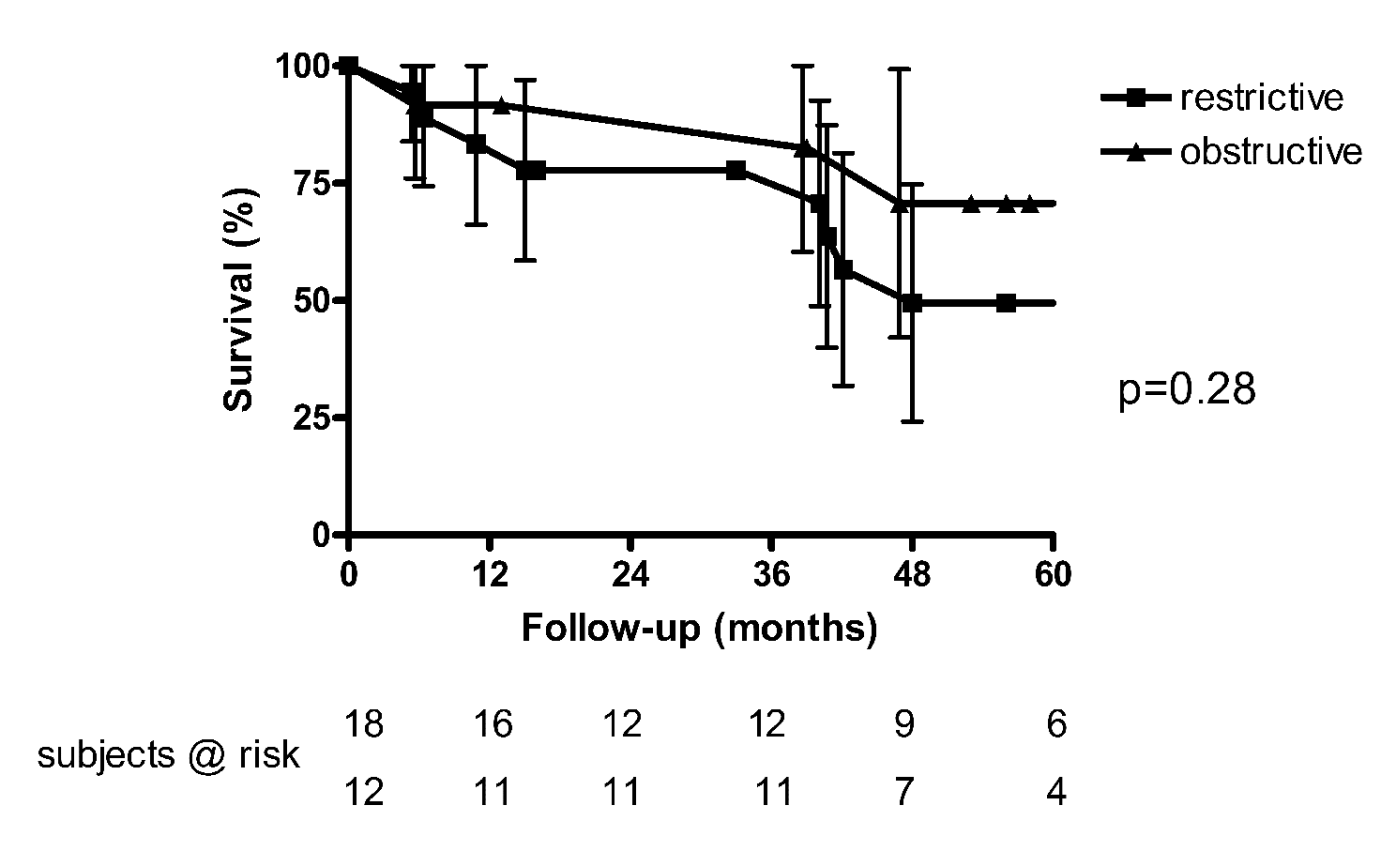
Kaplan-Meyer survival analysis: left versus right single lung transplantation

Age was distributed similarly in recipients with restrictive and obstructive disease (61 ± 5 versus 56 ± 9, *p* = 0.10). Recipients with restrictive lung disease had a higher LAS (median 45 versus 34, *p* < 0.001). Survival tended to be superior in the group with obstructive lung disease (1-year-survival: 92 versus 83%, 5-year-survival: 70 versus 49%, *p* = 0.28; Fig. 4). Post-transplant FEV1 was superior in recipients with restrictive lung disease (70 ± 15% versus 57 ± 11%, *p* = 0.02). Five of 9 surviving COPD-patients exhibit no or mild CLAD (no CLAD: *n* = 4; CLAD stage I: *n* = 1), while 4 COPD-patients have advanced CLAD (CLAD stage II: *n* = 2; CLAD stage III: *n* = 2). All fibrosis-patients have no or mild CLAD (CLAD stage 0: *n* = 8, CLAD stage I: *n* = 1).

## Discussion

Double lung transplantation (dLTx) is associated with a better postoperative functional capacity when compared with sLTx [6, 7]. However, a clear survival benefit conferred by dLTx has not unequivocally been documented [7, 8]. Can we therefore offer dLTx to all recipients? Or should we rather perform sLTx whenever possible in face of severe organ shortage and associated waiting list mortality?

Single center studies and registry-based studies have reported periprocedural and long-term outcomes after sLTX and dLTx. However, no prospective randomized trials have ever been performed to clearly document the individual merit of both procedures. Nevertheless 75% of lung transplantations are nowadays performed as dLTx [9]. 

Pulmonary fibrosis is as restrictive pulmonary disease is frequently associated with secondary pulmonary hypertension. SLTx for pulmonary fibrosis will therefore lead to preferred ventilation of the graft and preferred perfusion of the graft resulting in an optimal ventilation perfusion match. Accordingly sLTX was considered to be an optimal procedure for fibrosis [10]. Meyers reported the first larger cohort of recipients with pulmonary fibrosis and did not observe a survival difference for sLTx vs. dLTx [11]. In a more recent analysis with pooled data from the United Network for Organ Sharing (UNOS) database a better graft survival was documented with dLTx for recipients with pulmonary fibrosis [12]. In contrast a current study based on pooled data of the Scientific Registry of Transplant Recipients employed propensity score matching to compare sLTX and dLTx. Long-term-survival up to ten years was similar in both groups (*n* = 466 in each group). A trend towards reduced rate of posttransplant renal failure and reduced hospital length of stay was observed in sLTx-recipients [13]. 

SLTX for patients with COPD / emphysema is usually technically simple and rarely requires cardiopulmonary bypass. However, air trapping in the native lung may cause mediastinal shift and impaired ventilation-perfusion match. Thus volume reduction of the contralateral lung has been suggested to overcome this clinical dilemma [10]. If used with this precautions sLTx may lead to satisfactory long-term results, particularly for the elderly recipient. Thabut used the ISHLT database to analyse recipients between 1987 and 2007 worldwide. He documented a survival benefit of dLTx for recipients < 60 years [14]. The study by Schaffer et al. mentioned above, which employed the UNOS database, did not find a survival difference between sLTx and dLTx for COPD-patients [12]. 

The LAS-system was implemented in the USA in 2005 and in 2011 in Germany. With this allocation model decreasing mortality on the waiting list was observed in both countries [1, 15]. . While in Germany up to every fifth patient died on the waiting list before the advent of the LAS-system, mortality was reduced since then by 25% [1]. Nevertheless death on the waiting list is still an issue. A recent analysis based on the UNOS database documented that in only 43% of donors for sLTx, both lungs were used [16]. Thus centers should rethink their individual donor profiles. While an earlier analysis shed a critical light on lung splitting and sLTx [17], a very recent editorial by Ramos identified a decreased risk for death on the waiting list when patients with COPD or fibrosis were listed for sLTx without a compromised posttransplant outcome [9]. 

Lung twinning, i.e. two sLTx from one lung donor, was first reported by Haydock in a multi-center-study [18]. This strategy may help to overcome donor lung shortage, but exposes the second donor lung to prolonged ischemic time since both single lung transplantation may only rarely be performed at the same time in different operating rooms by different teams in the same transplant center. Prolonged cold ischemia may in turn lead to ischemia/reperfusion injury. Improvements in lung preservation, surgical technique and perioperative care have helped to reduce the reduce the incidence of ischemia/reperfusion induced primary graft failure from 30 to 15% or less. Nevertheless ischemia/reperfusion injury remains a significant cause of early morbidity and mortality after lung transplantation.

Does the lung twinning concept turn the second twin (i.e. the low-risk patient with lower LAS) into a high-risk patient due to increased risk of ischemia/reperfusion injury? Sommers analyzed differences between lung twin pairs in the Pittsburgh transplant program and observed impaired early graft function associated with left-single-lung-recipients, pulmonary hypertension and cardiopulmonary bypass [19]. In contrast, Glanville documented that prolonged ischemia for the second lung did not induce early graft dysfunction [20]. Snell reported the largest single-center-experience of lung twinning with 38 pairs of recipients [21]. This Australian group did not observe different outcomes between first and second twins. However this group reported an inferior intermediate outcome of left-single-lung-recipients - primarily related to increased mortality from airway complications [21]. These observations were supported by Smits from Eurotransplant, who analyzed the outcome of 90 lung twin pairs operated in 16 European centers [5]. In this analysis more fatal complications were observed in recipients receiving a left-sided sLTx. Outcome was particularly worse if the retrieval center was different from the transplanting center (1-year-survival: right sLTx 92% / left sLTx 62%, *p* = 0.04).

Our results with lung twinning support the findings of prior studies that lung twinning can be performed safely despite of prolonged ischemic time for the second lung. Primary graft dysfunction was not observed in our twin patient cohort, cumulative ventilation times and ICU lengths of stay were similar for first and second twins. Survival of our twin cohort was similar when compared with all standard sLTx and dLTx, which had been performed in the same era. Our data document that also challenging transplantations in high-LAS patients (*n* = 6) or ECMO (*n* = 3) may be performed in this context without survival difference between first twin (high-risk-patient) and second twin (low-risk patient). Intermediate-term outcomes are comparable with outcomes in the ISHLT registry. Of interest, no survival difference was observed for left versus right sLTx in our patient cohort. Maybe this finding could be attributed to the fact that all retrievals were performed by our center and no airway complications were observed. The left lung was used in 81% of the cases for patients with pulmonary fibrosis. This strategy is based on the observation that a lung allograft transplanted in a left chest of a recipient with pulmonary fibrosis could expand to its own size [22]. Accordingly, an excellent postoperative FEV1 of 70% was observed in our recipients with pulmonary fibrosis.

As matter of fact the first lung - typically (i.e. 75%) the left side - was allocated to recipients with pulmonary fibrosis at a higher LAS (median LAS 45). The remaining second lung allograft – typically the right lung – was accordingly allocated to patients with lower LAS (median 34) with COPD (56%), pulmonary fibrosis (31%) or bronchiolitis obliterans syndrome (13%). This strategy helped to minimize waiting list time - even though waiting list times were still similar when compared with double lung transplants (median 145 versus 283 days, *p* = 0.76). Waiting list time of the second twins tended to be longer (median 964 vs. 283 days, *p* = 0.056) - we speculate that waiting time for a double lung transplant allograft would have been significantly longer for these low LAS recipients.

We conclude that stringent use of sLTX - i.e. (almost) always for pulmonary fibrosis and if suitable for COPD - may expand the donor pool and allows lung twinning. Such concept can lead to encouraging intermediate-term outcomes and may help to further reduce waiting list mortality in the LAS-era.

## Data Availability

No datasets were generated or analysed during the current study.
